# Patient-Reported Outcome of Surgical Treatment of Nerve Entrapments in the Proximal Forearm

**DOI:** 10.4061/2011/727689

**Published:** 2011-09-11

**Authors:** Birgitta Svernlöv, Göran Nylander, Lars Adolfsson

**Affiliations:** ^1^Department of Plastic Surgery, Hand Surgery and Burns, Linköping University Hospital, 581 85 Linköping, Sweden; ^2^Department of Clinical and Experimental Medicine, Faculty of Health Sciences, Linköping University, 581 85 Linköping, Sweden; ^3^Department of Orthopaedics and Sports Medicine, Linköping University Hospital, 581 85 Linköping, Sweden

## Abstract

The outcome of decompression for long-standing symptoms of nerve entrapments in the proximal forearm was investigated in a retrospective study of 205 patients using a self-assessment questionnaire, 45 months after the operation. The questionnaire consisted of visual analogue scale recordings of pre- and postoperative pain during rest and activity, questions about remaining symptoms and appreciation of the result and the Disabilities of Arm, Shoulder and Hand form (DASH). Altogether, 59% of the patients were satisfied, 58% considered themselves improved, and 3% as being entirely relieved of all symptoms. Pain decreased significantly (*P* = 0.001). There was a significant correlation between preoperative duration and patient perceived post-operative pain. Preoperative pain was a chief complaint, and pain reduction appears to be the principal gain of the operation. Although the majority of the patients benefited from the operation, a substantial proportion was not satisfied. There is apparently room for improvement of the diagnostic and surgical methods applied in this study.

## 1. Introduction

Entrapment neuropathies are common conditions in the upper extremity. The most common is the carpal tunnel syndrome (CTS) for which diagnosis and treatment usually are uncontroversial. 

 There are, however, other nerve affections around the elbow that may be more difficult to diagnose. The most frequently involved nerves are believed to be the median and ulnar nerves [1]. Radial nerve involvement is a less encountered condition [2], estimated to represent about two percent of all proximal nerve entrapments in the upper limb [3]. Sometimes, multiple nerve compressions coexist [4–7].

Most nerve entrapments are hypothesized to be caused by external compression. Anatomic structures incriminated include fibro-osseous or fibromuscular tunnels or a muscle which the nerve courses through. In some cases, repetitive stress from overuse may induce muscular changes causing further narrowing which in turn may lead to nerve compression [4, 7]. The diagnosis of nerve entrapment relies heavily on history and physical examination. Subjective symptoms may be paraesthesia and/or numbness in the area of the nerve distribution, pain during activity and rest, and not infrequently nocturnal pain. Electrodiagnostic examinations are sometimes useful in confirming the diagnosis, but, when found negative, the condition cannot be ruled out [8–10]. Some authors maintain that provocative clinical testing alone is sufficiently sensitive for the diagnosis of nerve entrapments/compressions in the upper extremity [9, 11].

The initial treatment is usually conservative, including avoidance of provocative activities, immobilization in a splint, physical therapy, and patient education. When this fails, operation is often called for [1, 10, 12–14]. 

The most frequently applied surgical method is decompression [15–17].

In the absence of an established consensus on measuring the outcome of treatment, the efficacy of operation often has to be evaluated based on reduction of subjective symptoms. 

The aim of this study was to assess subjective outcome of decompression surgery in a patient cohort with long-standing symptoms where conservative treatment had been insufficient. 

## 2. Material and Methods

From January 2000 to April 2006, 217 consecutive adult patients with clinical signs of entrapment neuropathy in the proximal forearm were operated upon. All patients had long- standing pain over the alleged compression sites, both at rest and during activity, which had not been sufficiently controlled by physiotherapy and pharmacological treatment. Symptoms reported also included paraesthesia and/or numbness along the nerve distributions and disturbed sleep due to pain or numbness in the affected arm. At clinical examination all patients had tenderness at palpation over the suspected compression sites and occasionally weakness in the muscles innervated by the affected nerve. There were no signs of cervical radiculopathy or pathology in the elbow joint.

Two hundred and five patients (94%), 97 men and 108 women, were available for followup at an average of 45 months (range 9–87) after operation. Average age at operation was 46 years (range 18–77). Preoperatively, the mean symptom duration was 33 months (range 3–300). The dominant arm was involved in 137 of the cases. The diagnoses were distributed as follows: 76 patients with affection of the median nerve (pronator syndrome, PS), 33 patients with affection of the radial nerve (radial tunnel syndrome, RTS), 72 patients with affection of the ulnar nerve (cubital tunnel syndrome, CuTS), 20 patients with unilateral involvement of both the median and ulnar nerves, three patients with unilateral median and radial nerve symptoms, and one patient with unilateral ulnar and radial nerve involvement.

The followup consisted of a self-administered questionnaire and the Disabilities of Arm, Shoulder and Hand form (DASH) [18] sent out by mail to all patients with a prepaid return envelope, a cover note, and an informed consent form for signature to participate in the study. The participants were reassured of the confidential nature of the study. 

The questionnaire was designed by an independent investigator (BS) and included questions regarding current symptoms, such as paraesthesia and/or numbness, disturbed sleep, and pain at rest and during activity. The intensity of pain, both preoperative and current, was assessed by using two (rest and activity pain) visual analogue scales (VAS) (100 mm; 0, no pain, through 100, most pain imaginable) [19]. Furthermore they were asked to report their perceived functional status after operation by answering the following question: “How do you assess the function of your arm today as compared to before the operation?” on a four-point scale with the following alternatives: completely recovered, improved, unchanged, or worse [20]. In addition, patients were asked to rate their satisfaction with the result of the operation on a four-point scale, with the following alternatives: very satisfied, satisfied, dissatisfied, and very dissatisfied, and whether they would undergo the operation again, based on what they know today with the following alternatives: yes, maybe, and no [12, 21–25].

The results of the two pain ratings, VAS recordings (at rest and during activity), both before operation and at the long-term followup, were added, and the means of the total pain scores (TPSs) were used for further calculations.

A reviewer who was not involved in the operation carried out the study, which was approved by the regional ethical review board.

### 2.1. Surgical Procedures

Senior hand surgeons performed the operations. All operations were performed with the patient under general anaesthesia. After exsanguinations a tourniquet inflated to 60–70 mmHg above systolic blood pressure was used. In connection with operation at the proximal elbow, 78 (38%) simultaneous carpal tunnel releases (CTRs) were performed. Of these, 72 (92%) were in patients operated for pronator syndrome and six (3%) for cubital tunnel syndrome. No complications were recorded during the operations.

#### 2.1.1. Median Nerve

A curved or S-shaped incision was made at the volar, medial aspect of the forearm, starting at the elbow flexor crease and running distally 6-7 cm. The median nerve was followed distally under or through the pronator teres muscle, depending on the anatomic variations. The nerve was followed with perineural dissection until the superficial flexor arch, which was sharply divided, confirming that the nerve was free from any kind of direct external pressure. The anterior interosseous nerve branch was identified.

#### 2.1.2. Radial Nerve

In the majority of cases, the radial nerve was approached from the dorsal aspect using a straight 8 cm incision. The brachioradialis and the extensor carpi radialis longus muscles were separated, the medial fascial edge of the extensor carpi radialis brevis was divided as necessary, and the arcade of Frohse was identified. The superficial branch, the muscular branches, and the deep branch—the posterior interosseous nerve (PIN)—were identified. The PIN was then followed to the proximal aponeurotic edge of the supinator muscle, which was sharply incised 2-3 cm, until the PIN was released from any tendinous or fibrotic structures. Some surgeons preferred a volar approach and used a curved incision on the volar, radial aspect of the forearm, extending from the flexor crease and 8 cm distal. The incision in the supinator muscle was similar regardless of the approach. In no case was the distal part of the supinator muscle incised.

#### 2.1.3. Ulnar Nerve

The skin incision extended from the medial epicondyle 5 cm distally and 6 cm proximally. One or two sensory nerves were frequently found under the subcutaneous fat immediately distal to the epicondyle and were protected. The fascia was then incised directly over the ulnar nerve distally to the cubital tunnel and the muscle fibres separated. The distal part of the ligament over the cubital tunnel and both the outer and deep fascia of flexor carpi ulnaris were divided distally a few centimetres. Proximal to the medial epicondyle the ulnar nerve was followed 7-8 cm to ascertain no compression from Struthers' arcade. A portion of the roof of the cubital tunnel, proximal to the epicondyle, was left intact to avoid subluxation of the nerve. A thorough examination was done to ensure that there was enough space for the nerve in the rest of the cubital tunnel. No transpositions were made.

#### 2.1.4. Postoperative Treatment

All wounds were covered in soft dressings, and the patients were instructed to use the operated extremity in normal everyday activities immediately after operation. Heavy work or lifting was not allowed for six weeks. In the postoperative rehabilitation program, all patients were followed according to our structured care plan, meaning that a surgeon saw them at least once after the operation or, if needed, until they were back at work or back to their normal activities. All patients were seen by a physiotherapist and instructed in nerve gliding exercises, scar management, and to monitor possible pain and mobility, as well as by an occupational therapist, if necessary, for oedema reduction and/or treatment of scar hyperaesthesia if present. 

Data were analysed using STATISTICA v 9.1 StatSoft, Inc. (http://www.statsoft.com/). Paired *t*-test was used to test differences between the pain ratings (VAS) before operation and at the followup. One-way ANOVA was used to test the difference in pain score, and in post-operative DASH, among the means of the four nerve groups before and after operation. Pearson's product-moment correlation was used to test the existence of a relationship between the variables TPS (VAS) pre- and postoperation and duration of symptoms before operation and between TPS after the operation and DASH. Spearman Rank Order Correlation was used to test any relationship between the post-operative TPS and the variables subjective reported outcome, and satisfaction (both assigned numbers from 1 through 4, 1 being the highest score), and between subjective reported outcome and post-operative DASH. Chi-square test was used to test the difference in group proportions related to diagnosis.

 Logistic regression was used to test any relationship between the reported outcome and neurophysiologic reading, as well as simultaneous CTR, and time to followup.

*P* values <0.05 were considered significant. A 95% confidence interval was calculated. A professional statistician assisted in the analyses.

## 3. Results

The different nerve decompression groups were similar with respect to demographic data except for gender, where the ulnar nerve group had a male predominance while in all other groups there were more women (*P* = 0.008). The groups with affection of the median and radial, and the ulnar plus radial nerves, respectively, were excluded from further statistical analysis due to insufficient sample sizes. Accordingly, 201 patients comprise the cohort described in the following analyses (Table 1). 

Nerve conduction studies were performed in 131 (65%) of the patients, in whom the suspected clinical diagnosis was confirmed in 96 (73%). There was a significant difference between the groups in the outcome of the neurophysiologic examination where pathological readings were more common in the ulnar nerve group (*P* < 0.001) (Table 2). No relationship was found between the patient-reported outcome and a positive neurophysiologic reading or simultaneous CTR or time to followup.

**Figure 1:**
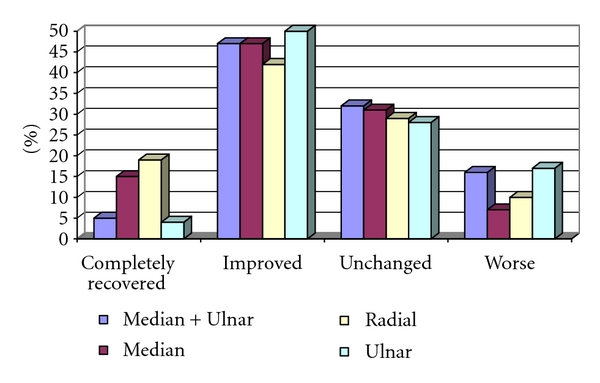
Patient self-reported outcome.

The reported subjective outcome after the operation showed that 21 patients (11%) considered themselves completely recovered, 91 patients (47%) improved, 57 patients (30%) unchanged, and 23 (12%) worse. There was no significant difference in perceived improvement between the different nerve groups (Figure 1). 

For the entire cohort, the categories “completely recovered,” “improved,” “unchanged,” and “worse” corresponded to a postoperative mean DASH score of eight, 31, 45, and 60, respectively. There was a significant correlation between the patient-reported outcome and postoperative DASH in the whole material and in three of the groups (*r*_*s*_ = 0.55, *P* < 0.05), indicating that those who scored high on the DASH also reported a worse outcome. There was also a positive correlation between patient-reported outcome and postoperative TPS in all of the four nerve groups as well as in the whole material (*r*_*s*_ = 0.62, *P* < 0.05), indicating that those who rated themselves high on the TPS also reported a worse outcome (Table 3).

**Figure 2:**
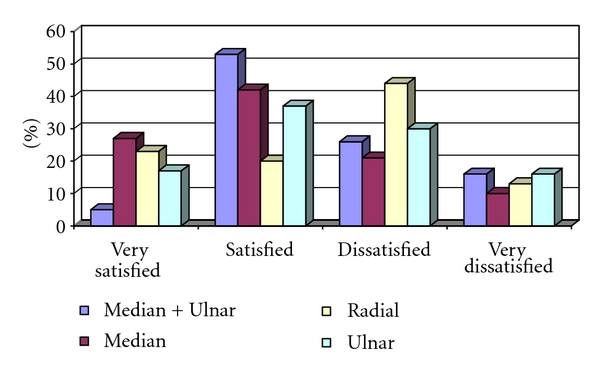
Patient-rated satisfaction.

Recordings of patient satisfaction showed that 40 patients (21%) were very satisfied, 73 patients (38%) were satisfied, 54 patients (28%) were dissatisfied, and 25 patients (13%) were very dissatisfied with the result of the operation. There was no significant difference between the nerve groups (*P* = 0.064) (Figure 2).

There was a significant relationship between satisfaction and post-operative TPS in the entire cohort and in three of the groups (*r*_*s*_ = 0.56, *P* < 0.05), demonstrating that those who rated themselves high on the total pain score also were less satisfied (Table 3). 

A significant relationship was also found between satisfaction and DASH in the entire cohort as well as in three of the groups (*r*_*s*_ = 0.43, *P* < 0.05), indicating that those who scored high on DASH also were less satisfied (Table 3). 

There was a significant relationship between the postoperative TPS and DASH in all the different nerve groups as well as in the whole cohort (*r* = 0.70, *P* < 0.05), indicating that those who rated themselves high on the total pain score also scored high on the DASH (Table 3). 

There was a statistically significant association between duration of symptoms and worse outcome as assessed by post-operative TPS in the whole cohort (*r* = 0.25) (*P* = 0.002).

One hundred and thirty-four patients (68%) answered that they would consider undergoing the operation again, 36 patients (18%) were indecisive, and 26 (13%) answered negatively. There was no difference between the nerve groups.

Regarding current symptoms, five (3%) of the subjects considered themselves to be completely relieved of all their preoperative symptoms, 11% of their previous paraesthesia or numbness, and 24% were completely relieved of any sleep disturbance. There was no significant difference between the nerve groups. 

The results from the two VAS pain ratings, rest pain and activity pain, and the TPS showed a significant decrease after operation, compared to before in the whole material *P* = 0.001 and *P* = 0.001, respectively (Table 4). Sixty-eight percent of the patients had less pain at rest, 70% less symptoms in connection with activity. 

There were no significant differences in TPS between the groups neither before nor after the operation. 

The mean postoperative DASH score was 35.29 (24.04) points for the whole patient population, for the median plus ulnar group 49.35 (27.44) points, for the median group 33.63 (24.77) points, for the radial group 26.31 (19.60) points, and for the ulnar group 36.95 (22.14) points.

## 4. Discussion

In our relatively large patient population, with long-standing symptoms of nerve entrapment in the proximal forearm, refractory to conservative treatment, we found that a total of 58% of the patients considered themselves to be completely recovered or improved by nerve decompression, although only five (3%) considered themselves to be completely relieved of all their preoperative symptoms. According to the results from the self-reported pain VAS recordings, there were significant decreases of pain in about 70% of the patients at the followup, which appears to be the main benefit of the operation.

Townshend and collaborators [25] stated that subjective outcome scores were more sensitive to change than traditional physical measures and that physical examination had little usefulness for predicting postsurgical functional limitations, symptoms, and/or satisfaction. 

Fifty-nine percent of the patients were very satisfied or satisfied with the result of the operation. 

Numerous factors may contribute to an unfavourable outcome and according to Ruchelsman and coworkers [26] one of which is unrealistic patient expectations. Therefore, patients with severe and chronic neuropathies should be enlightened about the limitations of surgical treatment. If the goals of the treatment are not fully defined preoperatively, patients who only experience pain relief without significant improvement of other symptoms may regard the operation as a failure [26].

The reason why a substantial proportion of the patients in our study did not experience a satisfactory improvement may relate to the severity and duration of symptoms, as we found a significant relationship between total pain score after the operation and the duration of symptoms before the procedure. The importance of symptom duration as a prognostic factor that may significantly influence the result of surgery was highlighted in a recent study of treatment of proximal forearm neuropathy [27]. 

Previous studies using patient satisfaction as the primary outcome measurement are few and based on small study samples. In a study by Bolster and Bakker [21], 92% of the 12 patients reported, at followup on average 10 months, satisfaction with the result of decompression of the radial nerve, and Tomaino and coworker [24] reported a 100% very satisfied or satisfied result 38 months after operation in their study of 16 patients who underwent in situ ulnar nerve release with medial epicondylectomy. 

We found only one other study in the literature that used subjective improvement as an outcome measurement after ulnar nerve transposition, by Novak and colleagues [12]. In this study, a telephone interview was used to assess subjective improvement after the transposition. They reported that 61 (75%) of the 81 patients considered themselves to be improved at the followup after 37 months. 

The results presented in these reports are superior to the findings in our study and could possibly be explained by the dissimilarities in group sizes, variations of surgical methods, follow-up times, and patient selection. Another explanation might be that in the present study the surgeons were not involved in the assessments. 

Novak et al. [12] reported that seven (8%) of their 81 patients considered themselves to be worse, and, in our study, 12% of the patients reported a worsening of their status, the majority having ulnar nerve affliction. Simple decompression surgery remedies only one aspect of the problem, that is, the direct compression on the nerve, but friction from surrounding structures or tension due to impaired gliding possibility may still exist, accentuated with elbow flexion [12]. Residual symptoms of numbness and weakness following surgical treatment of chronic ulnar neuropathy have been found common, possibly due to irreversible neural changes [26]. However, operation might still be indicated as the only treatment with the possibility to improve other complaints, such as pain and tenderness at the compression site and dysaesthesias in the hand [26]. 

Accordingly, it appears that patients with ulnar nerve entrapment deserve special attention when surgical treatment is considered. The difficulties in the treatment of ulnar entrapment have been recognised by others, and some authors advocate transposition of the ulnar nerve as the primary treatment [28]. Concomitant disorders, for example, a simultaneous medial epicondylitis, may be another factor for an unsatisfactory result [8, 26].

Some patients in the radial nerve group were dissatisfied with the outcome, and one reason could be the coexistence of a tendinopathy at the lateral epicondyle, which was not resolved by the decompression. It has previously been recognised that it is not infrequent that patients with RTS may have concomitant lateral epicondylitis [13, 29, 30]. According to Sotereanos and coauthors, the incidence of coexisting maladies may be as high as 43% [23]. 

Sixty-eight percent of the patients in our study would definitely consider operative treatment again. This figure is lower than reported in other studies using the same question. In a study on cubital tunnel decompression Nathan and coworkers [22] reported that, at the followup ranging from one to 12 months, 98% of their 74 patients were positive to have the operation again if needed. In studies with somewhat longer followup, Novak and associates [12] found that 83% of the 81 patients would choose to have ulnar nerve transposition surgery again, and Tomaino and coauthors [24] reported that all of their 16 patients would undergo the operation for CuTS again. Bolster and Bakker [21] showed that 83% of the 12 patients who underwent operation for RTS would do so again, and in 1999 Sotereanos and associates [23] completed a study of surgical treatment for RTS and found that 68% of the 28 patients would, at the followup on average 28 months, consider having the operation again. 

The ability of electrodiagnostic examination to confirm a compressive neuropathy in the forearm is unclear. This uncertainty was the reason why a substantial proportion of the patients in this study were not subjected to this test. According to Mackinnon and Novak [31], the nerve conduction part of the electrodiagnostic examination has some limitations; for example, symptoms such as pain and paraesthesia cannot be objectified. Furthermore, nerve problems occurring very distally or very proximally in the extremity are difficult to assess [31]. 

We were not able to establish any relationship between preoperative pathologic neurophysiologic findings and patient self-reported outcome in any of the nerve groups. LeRoux and associates [17] did not find any relationship between preoperative electromyography or/and nerve conduction velocity (NCV) and the outcome in their study of 51 patients who showed symptomatic improvement in 80% following simple decompression of the ulnar nerve. They further stated that neither pre- nor postoperative NCV are reliable predictors of outcome. Mackinnon and Novak [31] and Goldfarb [8] declared that electrodiagnostic testing is often negative in CuTS, which is why clinical diagnosis remains the gold standard. Electrodiagnostic examination is frequently normal in RTS according to some authors [7, 8, 13]. 

We realize that, since the DASH score was recorded only at followup, it is not useful in evaluating improvement. DASH, in this case, was introduced to illustrate the patient-reported function in relation to the other applied instruments, the subjective outcome score, the total pain score, and the satisfaction score. We found a high correlation between all the used assessment instruments. We also believe that the well-known DASH score aids in interpreting the findings of the other scores. 

The strengths of this study are that the patients were consecutively included, the high participation rate (94%), and that an independent researcher designed and conducted the followup. Furthermore, the surgical procedures were performed by a handful of surgeons working in the same setting and using the same protocol. This large sample of patient-reported post-operative results offers valuable insight concerning patient subjective status four years after nerve decompression in the proximal forearm.

Limitations of the present study comprise the absence of a control group, retrospective recall of preoperative pain, and the use of a nonvalidated subjective self-reported questionnaire. Another limitation is that the patient may have difficulties differentiating between symptoms that are strictly associated with the nerve in question and other disorders present during the follow-up period.

## 5. Conclusions

In conclusion, in a group of patients with long-standing entrapment neuropathy of the forearm, refractory to conservative treatment, a significant pain reduction was found in approximately 70%. Nearly 60% were satisfied with the results, and 68% would consider operation again. This can be looked upon as a treatment that provides help to a majority of patients where other options have failed; on the other hand, for a large proportion of patients in this study, the operation may be regarded as unnecessary. This underlines the need for improved diagnostic accuracy, and algorithms for patient selection are obvious, and there is a lack of high-quality studies with prospective design.

## Figures and Tables

**Table 1:** Demographics. Study group. Figures represent mean, SD, and absolute numbers.

	Median + ulnar	Median	Radial	Ulnar
	*n* = 20	*n* = 76	*n* = 33	*n* = 72
Gender M/F	6/14	34/42	11/22	45/27
Age (years)	43 (11.0)	48 (11.0)	46 (8.9)	45 (13.7)
Dominant arm	17	51	23	43
Duration (months)	49.7 (56.3)	43.1 (53.4)	34.5 (27.5)	31.9 (29.6)
Follow-up time (months)	37 (22.5)	45 (59.4)	43 (55.2)	49 (23.0)

**Table 2:** Neurophysiological findings of the examined patients (*n* = 131).

	Median + ulnar	Median	Radial	Ulnar
	*n* = 17	*n* = 60	*n* = 5	*n* = 49
Pathologic	10	38	3	45
Normal	7	22	2	4

**Table 3:** Relationship between total pain score (VAS) and DASH, and factors related. All correlation coefficients (*r*) are significant at *P* < 0.05.

	Median + ulnar	Median	Radial	Ulnar
	*n* = 20	*n* = 76	*n* = 33	*n* = 72
DASH				
VAS (total score after operation)	0.86	0.74	0.76	0.56
Satisfaction	NS	0.49	0.58	0.28
Subjective outcome	NS	0.65	0.67	0.35

VAS (total score after operation)				
Satisfaction	NS	0.60	0.65	0.45
Subjective outcome	0.58	0.58	0.80	0.59

**Table 4:** The results from the pain VAS recordings (mm). Mean values, SD, and *P* values are presented.

	Median + ulnar	Median	Radial	Ulnar
	*n* = 20	*n* = 76	*n* = 33	*n* = 72
Pain at rest				
(0 months)	48.88 (22.79)	43.92 (27.93)	44.32 (23.56)	41.07 (20.58)
(45 months)	31.06 (24.86)	22.42 (25.08)	29.61 (22.87)	24.21 (20.20)
	*P* = 0.005	*P* = 0.0001	*P* = 0.003	*P* = 0.0001
Activity pain				
(0 months)	60.94 (18.17)	54.39 (24.13)	58.07 (21.99)	55.51 (18.81)
(45 months)	50.00 (25.05)	30.89 (26.89)	40.42 (23.78)	37.73 (24.37)
	N.S.	*P* = 0.0001	*P* = 0.004	*P* = 0.0001
Total pain score				
(0 months)	54.90 (16.88)	49.16 (24.60)	51.20 (20.87)	48.29 (17.85)
(45 months)	40.53 (23.76)	26.66 (25.24)	35.02 (22.59)	30.97 (21.46)
	*P* = 0.03	*P* = 0.0001	*P* = 0.002	*P* = 0.0001
